# Identification of appropriate reference genes for qPCR studies in *Staphylococcus pseudintermedius* and preliminary assessment of *icaA* gene expression in biofilm-embedded bacteria

**DOI:** 10.1186/1756-0500-7-451

**Published:** 2014-07-15

**Authors:** Evan C Crawford, Ameet Singh, Devon Metcalf, Thomas WG Gibson, Scott J Weese

**Affiliations:** 1 Department of Clinical Studies, Ontario Veterinary College, University of Guelph, 50 Stone Road, Ontario N1G 2 W1, Canada; 2 Department of Pathobiology and Centre for Public Health and Zoonoses, Ontario Veterinary College, University of Guelph, 50 Stone Road, Ontario N1G 2 W1, Canada

**Keywords:** qPCR, Canine, MRSP, Biofilm, Reference gene

## Abstract

**Background:**

Quantitative PCR is rapidly becoming the standard method for analyzing gene expression in a wide variety of biological samples however it can suffer from significant error if stably expressed reference genes are not identified on which to base the analysis. Suitable reference genes for qPCR experiments on *Staphylococcus pseudintermedius* have yet to be identified.

**Results:**

Three reference genes in *S. pseudintermedius* were identified and validated from a set of eight potential genes (*proC*, *gyrB*, *rplD*, *rho*, *rpoA*, *ftsZ*, *recA*, *sodA*). Two strains of *S. pseudintermedius* were used, and primer specificity and efficiency were confirmed and measured*.* Ranking of the genes with respect to expression stability revealed *gyrB*, *rho* and *recA* as the best reference genes. This combination was used to quantify expression of a single biofilm associated gene, *icaA*, in logarithmic, stationary and biofilm growth phases, revealing that expression was significantly upregulated in the biofilm growth phase in both strains.

**Conclusion:**

Three reference genes, *gyrB, rho* and *recA,* were identified and validated for use as reference genes for quantitative PCR experiments in *S. pseudintermedius*. Also, the biofilm associated gene *icaA* was shown to be significantly upregulated in biofilm samples, consistent with its role in biofilm production.

## Background

*Staphylococcus pseudintermedius* is a common commensal organism of canines, but is also one of the most common causes of opportunistic infections [1-3]. Recently, methicillin resistant *S. pseudintermedius* (MRSP) has emerged and disseminated internationally [4,5], with 2 major sequence types (ST68 and ST71) representing the variety of clinical infections in most regions [3]. One area of concern is the ability of this bacterium to produce biofilm something that might be an important virulence factor and complicate elimination of infections [6,7]. Expression of genes pertaining to initial bacterial surface adherence and intercellular adhesion following biofilm formation, such as microbial surface components recognizing adhesive matrix molecule (MSCRAMMs) which mediate cellular adhesion, and the intracellular adhesion (*icaADBC*) operon [8], reported to be at least partially responsible for biofilm formation, likely affect the *in vivo* behavior of this organism, including resistance to therapy [9,10]. While there is significant postulation regarding these factors, understanding of the expression of antimicrobial resistance and biofilm associated genes in *S. pseudintermedius* and the subsequent clinical implications is still poor.

Quantitative real-time PCR (qPCR) is increasingly employed to quantify gene expression. While it can be very sensitive and specific, there are numerous pitfalls in its application that can easily result in misleading and incorrect conclusions. On of the most frequent errors is a failure to confirm the constitutive expression of the reference genes used to measure the relative expression of genes of interest. Normalization of results in qPCR is vital to limit variability introduced by experimental conditions, sample preparation and analysis, and is one of the main underlying tenets of qPCR analysis. Selection of inappropriate reference genes can result in grossly incorrect conclusions owing to the miscalculation of gene expression. These and other reasons have prompted the development of minimum information for publication of qPCR experiments (MIQE) guidelines to ensure integrity, consistency and transparency of qPCR experiments, including standards for all aspects of experimental design, analysis and reporting [11].

Current recommendations suggest a minimum of three reference genes (ideally with M values below 1 for heterogenous samples), and the inclusion of additional genes as necessary to obtain a pairwise variation value < 0.15 [12]. This requires specific validation of candidate reference genes in the bacterium (and ideally strains) to be studied, something that is lacking for *S. pseudintermedius*. While multiple qPCR studies have been performed in various *Staphylococcus* spp [13,14], study and validation of reference genes in *S. pseudintermedius* is lacking, and it cannot be assumed that data from other staphylococci apply to this species.

The objectives of this study were to evaluate several potential reference genes in *S. pseudintermedius*, to identify the optimum gene or gene combinations for future qPCR expression studies and to evaluate expression of *icaA* using validated reference genes.

## Results and discussion

### RNA isolation

The modified protocol afforded good to excellent yields (mean yield 53.5 ± 23.4 μg, range 16.9 – 92.2 μg, concentration 639 ± 94 μg/μL), as well as good RNA purity and integrity (RNA Integrity Number mean 9.1 ± 0.4) (Table 1), though there was evidence of some mild contamination from purification reagents (260/230 < 2). qPCR was performed on all RNA preparations to confirm DNA elimination; quantification cycle numbers were at least 20 cycles lower for RNA samples than equivalent amounts of DNA.

**Table 1:** RNA quality and recovery

**Sample ID**	**Concentration (ng/μL)**	**260/280**	**260/230**	**Total RNA recovery (μg)**	**RIN**
A42 log A	616.2	2.15	1.09	61.6	9.6
A42 log B	771.4	2.15	2.27	77.1	n.t.
A42 log C	805.5	2.15	2.27	80.5	9.0
A54 log A	823.1	2.14	2.20	82.3	9.4
A54 log B	919.4	2.13	1.67	91.9	n.t.
A54 log C	921.6	2.14	2.03	92.2	9.4
A42 stat A	535.4	2.10	2.00	53.5	9.2
A42 stat B	449.4	2.02	1.95	44.9	n.t.
A42 stat C	493.0	2.05	1.66	49.3	8.8
A54 stat A	403.3	2.05	2.05	40.3	8.5
A54 stat B	630.1	2.13	1.89	63.0	n.t
A54 stat C	397.5	2.05	1.89	39.7	8.8
A42 BF A	732.4	2.12	2.17	36.6	9.4
A42 BF B	738.6	2.11	2.18	36.9	n.t
A42 BF C	887.3	2.12	2.19	44.4	8.4
A54 BF A	606.9	2.11	1.97	30.3	9.5
A54 BF B	337.9	2.06	1.74	16.9	n.t
A54 BF C	421.9	2.03	1.99	21.1	8.9

(n.t: not tested, log: logarithmic, stat: stationary, BF: biofilm samples).

The modified protocol for RNA and DNA extraction, namely the inclusion of lysostaphin (and Dispersin B for biofilm samples) in the initial lysis buffer resulted in substantial and reproducible increases in RNA and DNA yield during harvesting (results not shown). Dispersin B catalyzes the hydrolysis of polysaccharide intercellular adhesin (PIA), a major constituent of the extracellular matrix of *Staphylococcus spp.* biofilms. Incorporating this enzyme in the solution to recover the biofilm from a surface, and in the lysis buffer is thought to increase recovery by enzymatically degrading the extracellular matrix of biofilm, releasing adherent bacteria for recovery, and exposing them to the lysis solution. The ability of this simple method to yield an adequate quantity and quality of DNA from biofilm-embedded bacteria was an important finding and will facilitate future studies of gene expression in biofilms.

### Amplification specificity and determination of PCR efficiency of reference genes

Quantification cycle (Cq) for each reaction was plotted against the log of DNA concentration, and the slopes of the curves were used to calculate the PCR efficiency values, which ranged from 1.79 to 1.87, with excellent regression coefficients (Table 2). Despite the range in melting points of the qPCR products (Table 2), all primers amplified well under the conditions listed. Melt curve analysis showed a single melt curve for each target gene, and DNA agarose gel electrophoresis revealed a single peak for each product. Sequencing of the qPCR products matched the sequence of the desired target in all cases.

**Table 2:** **Reference and target gene efficiency determination and amplicon melting points, r**^**2 **^**values are regression coefficients for the curves**

**Candidate reference genes**	**Slope of the curve**	**r**^**2 **^**on the slope**	**PCR efficiency**	**Melting temperature (°C)**
*proC*	−3.78	0.997	1.84	80.0
*gyrB*	−3.85	0.998	1.82	79.5
*rplD*	−3.86	0.998	1.82	75.5
*rho*	−3.68	0.997	1.87	81.0
*rpoA*	−3.89	0.996	1.81	76.5
*ftsZ*	−3.85	0.998	1.82	79.0
*recA*	−3.83	0.998	1.82	81.0
*sodA*	−3.97	0.996	1.79	79.5
Target gene				
*icaA*	−3.92	0.995	1.80	80.5

### Comparison of qPCR reaction products

Melt curve analyses and gel electrophoresis were also performed on and compared between samples recovered from qPCR reactions performed on cDNA prepared using random hexamers, single gene specific primer and combination gene specific primer reverse transcript reactions; there were no differences in any of the products obtained from those obtained from qPCR or genomic DNA.

### Stability assessment and validation of reference genes

Relative expression levels (quantification cycle numbers) were entered into the Microsoft Excel (Microsoft Canada, Mississauga, ON) visual basic application geNorm [12], which calculated stability values (M values) for each gene (Table 3). GeNorm was also used to calculate normalization factors using combinations of the most stably expressed genes, and calculated the pairwise variation between these factors to identify the optimum number of reference genes to use (Table 4). The three genes with the lowest individual M values were used in combination (*rho, recA, gyrB*) for subsequent expression analysis.

**Table 3:** GeNorm gene stability (M) values

**Candidate reference genes**	**geNorm M value**
*proC*	0.701
*gyrB*	0.604
*rplD*	0.769
*rho*	0.595
*rpoA*	0.663
*ftsZ*	0.848
*recA*	0.599
*sodA*	1.150

**Table 4:** Pairwise variation in normalization factor for combinations of reference genes

**Number of genes used**	**Pairwise variation of normalization factors**
2 vs 3	0.119
3 vs 4	0.096
4 vs 5	0.082
5 vs 6	0.112
6 vs 7	0.107
7 vs 8	0.135

While stability of expression of reference genes is important, reference genes ideally also have a high PCR efficiency. The primers we identified had efficiency ranging from 1.79 to 1.84 (with a value of 2.0 representing 100% efficiency), and while the final three genes used for normalization did not have the highest efficiencies, the values are accounted for during normalization of qPCR expression data.

With respect to the identification of reference genes, these results are specifically only applicable to the two strains studied under the three growth phases sampled. However, the two strains that were studied comprised the two main international MRSP clones [3], suggesting that these genes will be suitable for broad studies of MRSP gene expression. The excellent stability of expression of these genes over the broad range of conditions studied also indicate that these targets will likely function very well as reference genes over a generally broad range of conditions.

### icaA expression

**Figure 1:**
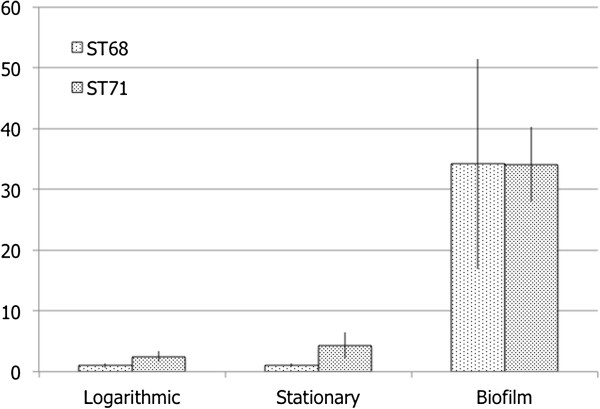
**Relative expression of *icaA*.**

Expression of *icaA* was significantly higher in the biofilm compared to logarithmic and stationary phases (p = 0.0093 (A42), p < 0.0001 (A54), Figure 1). Individual Tukey’s post-hoc p-values for individual comparisons were for logarithmic vs biofilm p = 0.015 (A42); p = 0.0001 (A54) and for stationary vs biofilm p = 0.015 (A42); p = 0.0002 (A54). There was no difference in expression level between logarithmic and stationary phases for either strain, individual Tukey post-hoc p > 0.9999 (A42), p = 0.8258 (A54).

These findings are unsurprising given the role of this gene in formation of polysaccharide intracellular adhesin, but it is noteworthy that such a profound alteration in expression was detectable. Further investigation of expression of this gene under other conditions and on a variety of surfaces, as well as studies of expression of other biofilm associated genes such as MSCRAMMs.

## Conclusion

Proper development of qPCR assays, including reference gene assessment, is a critical quality control step in the application of this tool. This study has identified a group of *S. pseudintermedius* reference genes, and used those reference genes to demonstrate a significant expression change in a biofilm associated gene. This information provides a vital background for the performance of gene expression studies in this increasingly important veterinary pathogen.

## Methods

### Bacterial strains and culture conditions

Two canine *S. pseudintermedius* strains were chosen, representing the two main international MRSP clones, sequence type (ST) 68 (strain A42) and ST71 (strain A54). These two strains were isolated from clinical infections, and were both previously classified as moderate biofilm formers using a polystyrene plate assay (unpublished data). Isolates were grown in tryptic soy broth supplemented with 1% (w/v) dextrose for all growth conditions. Samples were isolated as single colonies from streak plates prepared from −80°C freezer stocks on Columbia blood agar (Sheep blood), and incubated aerobically in a shaker at 37°C. Logarithmic phase growth was defined in a preliminary study (data not presented) as >1 and <6 hours of growth, and OD_600_ > 0.5 and <2.0; stationary phase growth was collected at >12 (12–24) hours, and OD_600_ > 2.2. For logarithmic and stationary samples, 10^9^ cells were harvested, centrifuged (13,000 × g for 30 seconds) to pellet the cells, then immediately processed to recover RNA as described below. Biofilm samples were produced by incubating glass Erlenmeyer flasks unshaken at 37°C for >48 (48–54) hours, at which time a visible film was present on the flask surface. The media was removed, and the flasks were washed twice with phosphate buffered saline (PBS, pH 7.4). Biofilm was harvested by incubating the flask with 10 ug/mL Dispersin B (Kane Biotech, Winnipeg, MB) a biofilm degrading enzyme isolated from *Aggregatibacter actinomycetemcomitans,* in PBS, shaking the flask for five minutes at room temperature (the visible film dispersed producing a turbid solution), and then pelleting by centrifugation (13,000 × g for 30 seconds) the cells present in the solution. The pellet of cells was immediately processed to recover RNA as described below.

### Primer design and determination of PCR efficiency

Eight candidate reference genes (Table 5) were evaluated using the validation software geNorm [12]. These genes had been examined in *S. epidermidis* and *S. aureus* in previous studies [13,14], and analogous sequences were identified in *S. pseudintermedius* for evaluation in this study. Primers were designed using a combination of GeneRunner software version 3.05 (Hasting Software, Inc.) and the National Centre for Biotechnology Information online primer designing tool (http://www.ncbi.nlm.nih.gov/tools/primer-blast/) using gene sequences for all strains of *Staphylococcus pseudintermedius* (strains ED99 and HKU10-03) available from Genbank (http://www.ncbi.nlm.nih.gov/genbank/).

**Table 5:** Candidate reference and target genes, primers and amplicon sizes

**Candidate reference genes**	**Function**	**Primer sequence (5’-3’)**	**Expected size (bp)**
*proC*	Pyrrolidine-5-carboxylate reductase	*proC*-F gccgaatacaaatgcgcacg	180
		*proC*-R aaaaatgcagggccacttcc	
*gyrB*	DNA gyrase B subunit	*gyrB*-F gcgtccgttgattgaagcg	240
		*gyrB*-R aacgtcacttgcaacatcgc	
*rplD*	50S ribosomal protein L4	*rplD*-F gcctaagaaaatgcgtcg	237
		*rplD*-R ccttctggtgttgtgattg	
*rho*	Transcription termination factor Rho	*rho*-F cacgtaaaagttgctgaattg	215
		*rho*-R cctgcttcgatatttctgg	
*rpoA*	DNA-directed RNA polymerase sigma factor	*rpoA*-F ctatcatcattaccaggtgc	231
		*rpoA*-R caaaatttcaacatcactgtc	
*ftsZ*	Cell division protein ftsZ	*ftsZ*-F gtccattcagtttcgaagg	254
		*ftsZ*-R catgattgttttaacgtcagc	
*recA*	Recombinase A	*recA*-F gcattaggtgtagatattgataac	228
		*recA*-R ggctgcagaaagtttacgc	
*sodA*	Superoxide dismutase	*sodA*-F cgcaaacttagacagcgtacc	227
		*sodA*-R caacaagccaagcccaacc	
Target Gene			
*icaA*	N-acetylglucosaminyl-transferase	*icaA*-F ttgcccaccttgtgcccacc	178
		*icaA*-R tgaggctgtagggcgttggga	

Primers for *icaA* were available from a previous study [15] and were validated for use in qPCR as described for the potential reference gene primers.

Pooled chromosomal DNA from the two strains under investigation (mixture of equal concentrations from each strain) was used to generate dilution series for PCR efficiency calculations and for amplicon melting point assessment. For efficiency determination, a six fold dilution series was prepared in triplicate. Twenty μL reactions containing from 100 ng to 0.1 pg of total DNA were prepared in 96 well plates (Bio-Rad) using the LightCycler 480 SYBR Green I Master qPCR reaction mixture (Roche Applied Science, Indianapolis, IN), following the manufacturer’s instructions. Primer concentration was 0.5 μM for each primer in the final reaction. A Bio-Rad C1000 thermal cycler with a CFX96 Real-Time System and Bio-Rad CFX Manager 2.0 software (Bio-Rad Life Sciences, Mississauga, ON) was used to run the following optimized thermocycling parameters: denaturation at 95°C for 10 minutes followed by 50 cycles of 10 seconds at 95°C, 30 seconds at 62°C, 30 seconds at 72°C, then a melting curve analysis running from 65 to 95°C with steps and measurements every 0.5°C. The thermocycler software calculated quantification cycle (Cq), efficiency and regression coefficients from the recovered data. Amplicon identity and primer specificity was confirmed by sequencing of the PCR products (fluorescent capillary Sanger method, Macrogen, Seoul, Korea), melt curve analysis and gel electrophoresis. qPCR reactions were tested over a range of primer concentrations and annealing temperatures and times to determine optimum reaction conditions.

### DNA isolation

Cells were collected from a logarithmic phase sample as described above, and processed using the High Pure PCR Template Preparation Kit (Roche Applied Science, Indianapolis, IN) following the manufacturer’s instructions with the exception that lysostaphin (Sigma-Aldrich, Oakville, ON) was added at 0.1 mg/mL in the initial lysis step. Concentration and purity at 260/280 nm was measured using a Nanodrop ND-100 spectrophotometer (Nanodrop Technologies Inc., Wilmington, DE), and the samples were qualitatively examined for shearing by DNA gel electrophoresis.

### RNA isolation

Each pellet of cells was resuspended in 2 mL PBS (pH 7.4), centrifuged to a pellet and the supernatant discarded. The pelleted cells were subsequently processed using the Qiagen RNeasy Mini kit (Qiagen, Germantown, MD), following the manufacturer’s instructions, with the following changes. The cells were initially resuspended in tris-EDTA buffer (pH 7.5) containing 15 mg/ml lysozyme (Sigma-Aldrich), 2 mg/mL proteinase K (Sigma-Aldrich), 25 μg/mL Dispersin B (Kane Biotech Inc., Winnipeg, MB) and 0.1 mg/mL lysostaphin (Sigma-Aldrich). All optional steps for additional washes or spins during the procedure were performed. Logarithmic and stationary phase samples were eluted from the purification column with two volumes of 50 μL sterile nuclease free water, biofilm samples were eluted with one volume of 50 μL (volumes chosen so as to obtain final RNA concentrations above 400 ng/μL). All samples were treated with the DNA*free* DNAse kit (Ambion, Austin, TX) as per the manufacturer’s instructions using a total of 2 units per reaction, added in two aliquots. A Nanodrop ND-100 spectrophotometer (Nanodrop Technologies Inc.) was used to measure RNA concentration and purity at 260/280 nm. Three samples of each RNA sample were measured and the measured concentrations were averaged. Representative samples were submitted for Bioanalyzer analysis (Agilent Technologies, Santa Clara, CA). Real-time PCR was performed using the efficiency determination protocol on RNA samples to confirm the absence of DNA using *gyrB* primers. RNA samples were stored at −20 to −80°C until further use.

### Reverse transcription/cDNA preparation

Two μg of total RNA was used in each 20 μL gene specific reverse transcription reaction using the Omniscript RT PCR kit (Qiagen), using 10 μM of each primer, 0.5 mM dNTP, 1 U reverse transcription enzyme, provided buffer diluted to 1x and the remainder as water. A mixture of forward and reverse primers for all nine genes was used. Reactions were incubated at 42°C for 60 minutes. Reverse transcription products were purified using the QIAquick PCR purification kit (Qiagen) as per the manufacturer’s instructions, eluted with 100 μL Qiagen EB buffer, and stored at −20°C until further use.

### qPCR for gene stability and *icaA*

The same protocol for efficiency testing was used to examine stability of potential reference gene and *icaA* expression, with the only change being that only 30 cycles were performed. In triplicate, cDNA produced from all three growth phases were subjected to qPCR (as described for the efficiency evaluation) for each candidate gene. In each 20 μL reaction, 5 μL of purified RT reaction product (equivalent to 100 ng of RNA prior to reverse transcription) was used. No template (negative; water) and DNA (positive; 100 ng DNA) controls were included in each run. No amplification was identified in negative controls. Intra-plate normalization was performed using the measured level of *icaA* in the positive control on each plate.

### icaA expression, statistical analysis

Expression of *icaA* was compared between the three growth phases for the two strains using the combination of three reference genes. Normalization factors, calibration factors and relative expression was calculated as per Hellemans et al. [16]. A one-way ANOVA with Tukey’s post-hoc test was perfomed to compare expression between growth phases. P-values < 0.05 were considered significant.

## Abbreviations

qPCR: Quantitative polymerase chain reaction; MRSP: Methicillin resistant *Staphylococcus pseudintermedius*; MSCRAMM: Microbial surface component recognizing adhesive matrix molecule; MIQE: Minimum information for quantitative PCR experiment; ST: Sequence type; OD: Ooptical density; w/v: Weight/volume; PBS: Phosphate buffered saline; cDNA: Complementary DNA; RIN: RNA integrity number; Cq: Quantification cycle.

## Competing interests

The authors declare that they have no competing interests.

## Authors’ contributions

EC performed the majority of the laboratory work, assisted by DM. Primer design was completed by EC and DM. The study was conceived by SW and AS, who also both participated in the experimental design. EC drafted the initial manuscript, and all authors were involved in editing and production of the final version. All authors read and approved the final manuscript.
